# 2-[4-(Methyl­sulfan­yl)phen­yl]naphtho[1,8-*de*][1,3,2]diaza­borinane

**DOI:** 10.1107/S1600536811014693

**Published:** 2011-05-07

**Authors:** Cathryn A. Slabber, Matthew P. Akerman, Ross S. Robinson

**Affiliations:** a Warren Research Laboratory, School of Chemistry, University of KwaZulu Natal, Private Bag X01, Scottsville, Pietermaritzburg 3209, South Africa

## Abstract

The title compound, C_17_H_15_BN_2_S, is one member in a series of diaza­borinanes featuring substitution at the 1-, 2- and 3-positions in the nitro­gen–boron heterocycle. The dihedral angle between the mean planes of the naphthalene and phenyl ring systems is 19.86 (6)°. In the crystal structure, two C—H⋯π inter­actions link the mol­ecules into sheets which lie parallel to the *bc* plane. There is a π–π inter­action between each pair of centrosymmetrically related sheets [centroid–centroid distance = 3.5922 (8) Å].

## Related literature

For the synthesis of the title compound, see: Slabber (2011 ▶). For the structures of related compounds and luminescence studies, see: Weber *et al.* (2009 ▶).
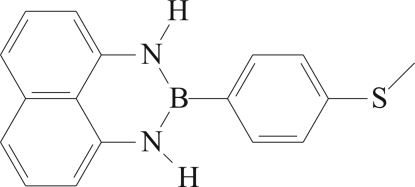


## Experimental

### 

#### Crystal data

C_17_H_15_BN_2_S
*M* *_r_* = 290.18
Monoclinic, 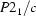

*a* = 13.7594 (6) Å
*b* = 9.0545 (3) Å
*c* = 12.7830 (5) Å
β = 113.411 (5)°
*V* = 1461.46 (10) Å^3^
*Z* = 4
Mo *K*α radiation
μ = 0.21 mm^−1^
*T* = 296 K
0.40 × 0.40 × 0.30 mm

#### Data collection

Oxford Diffraction Xcalibur 2 CCD diffractometer
Absorption correction: multi-scan *CrysAlis RED*; Oxford Diffraction, 2008 ▶) *T*_min_ = 0.919, *T*_max_ = 0.939
14782 measured reflections
4709 independent reflections
2774 reflections with *I* > 2σ(*I*)
*R*_int_ = 0.033

#### Refinement

*R*[*F* ^2^ > 2σ(*F* ^2^)] = 0.050
*wR*(*F* ^2^) = 0.149
*S* = 0.98
4709 reflections
199 parameters
H atoms treated by a mixture of independent and constrained refinement
Δρ_max_ = 0.42 e Å^−3^
Δρ_min_ = −0.49 e Å^−3^

### 

Data collection: *CrysAlis CCD* (Oxford Diffraction, 2008 ▶); cell refinement: *CrysAlis RED* (Oxford Diffraction, 2008 ▶); data reduction: *CrysAlis RED*; program(s) used to solve structure: *SHELXS97* (Sheldrick, 2008 ▶); program(s) used to refine structure: *SHELXL97* (Sheldrick, 2008 ▶); molecular graphics: *PLATON* (Spek, 2009 ▶); software used to prepare material for publication: *PLATON*, *SHELXL97* and *WinGX* (Farrugia, 1999 ▶).

## Supplementary Material

Crystal structure: contains datablocks I, global. DOI: 10.1107/S1600536811014693/lw2059sup1.cif

Structure factors: contains datablocks I. DOI: 10.1107/S1600536811014693/lw2059Isup2.hkl

Additional supplementary materials:  crystallographic information; 3D view; checkCIF report

## Figures and Tables

**Table 1:** Hydrogen-bond geometry (Å, °) *Cg*2 is the centroid of the C5–C9 ring and *Cg*3 is the centroid of the C11–C16 ring.

*D*—H⋯*A*	*D*—H	H⋯*A*	*D*⋯*A*	*D*—H⋯*A*
C2—H2⋯*Cg*3^i^	0.93	2.75	3.5178 (18)	141
C12—H12⋯*Cg*2^ii^	0.93	2.82	3.6122 (17)	144

Symmetry codes: (i) 
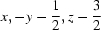
; (ii) 
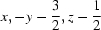
.

**Fig. 1.**
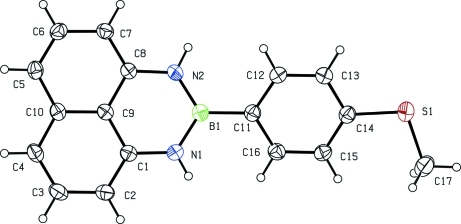
A view of the molecule showing the numbering scheme. Displacement ellipsoids are drawn at the 30% probability level.

## supplementary crystallographic information

### Comment

C_17_H_15_BN_2_S, is one compound in a series of diazaborinanes featuring substitution at the 1, 2 and 3 positions in the nitrogen–boron heterocycle. The dihedral angle between the mean planes of the naphthalene and the phenyl ring systems is 19.86 (6)°. The atoms N1, B1 and N2 lie at 0.053 (2) Å, 0.084 (2) Å and 0.017 (2) Å respectively above the mean plane of the naphthalene ring.

The H atoms attached to the N atoms are unavailable for hydrogen-bonding due to their positions in the molecule.

The supramolecular structure is determined by the C2—H2···Cg3(x, 0.5 - y, -0.5 + z) and the C12—H12···Cg2(x, -0.5 - y, 0.5 + z), where Cg3 is the centroid of the phenyl ring containing C11 and Cg2 is the centroid of the phenyl ring containing C8. These link the molecules into sheets which lie in the *bc* plane.

There is a π–π interaction between the centrosymmetrically related phenyl rings, containing C8, at (x, y, z) and (1 - x, -y, -z). The centroid-to-centroid distance is 3.5922 (8) Å, with a perpendicular spacing between the planes of 3.3883 (6) Å and a slippage of 1.193 Å.

### Experimental

To a solution of 1,8-diaminonaphthalene in toluene (4.11 mmol in 50 ml, 0.82 *M*) (Slabber, 2011) was added the 4-(methylthio)phenylboronic acid (4.11 mmol) in one portion. The round-bottomed flask was equipped with a Dean and Stark trap, and the solution was stirred and heated at 110°C for 3 h. The solvent was removed *in vacuo* and column chromatography of the crude solid on silica eluting with CH_2_Cl_2_ yielded yellow crystalline material in a yield of 65%. Crystals suitable for X-ray diffraction analysis were grown from CH_2_Cl_2_ at room temperature.

### Refinement

H atoms were treated as riding atoms with C—H(aromatic), 0.93 Å, with *U*_iso_ = 1.2*U*_eq_(C) and C—H3 (methyl), 0.96Å, with *U*_iso_ = 1.5*U*_eq_(C). H atoms attached to N atoms were located on a difference map, and allowed to refine.

### Crystal data

**Table:**

C_17_H_15_BN_2_S	*F*(000) = 608
*M**_r_* = 290.18	*D*_x_ = 1.319 Mg m^−^^3^
Monoclinic, *P*2_1_/*c*	Mo *K*α radiation, λ = 0.71073 Å
Hall symbol: -P 2ybc	Cell parameters from 5105 reflections
*a* = 13.7594 (6) Å	θ = 2.8–32.0°
*b* = 9.0545 (3) Å	µ = 0.21 mm^−^^1^
*c* = 12.7830 (5) Å	*T* = 296 K
β = 113.411 (5)°	Prismatic, yellow
*V* = 1461.46 (10) Å^3^	0.40 × 0.40 × 0.30 mm
*Z* = 4	

### Data collection

**Table:**

Oxford Diffraction Xcalibur 2 CCD diffractometer	4709 independent reflections
Radiation source: fine-focus sealed tube	2774 reflections with *I* > 2σ(*I*)
graphite	*R*_int_ = 0.033
Detector resolution: 8.4190 pixels mm^-1^	θ_max_ = 32.1°, θ_min_ = 2.8°
ω scans	*h* = −19→20
Absorption correction: multi-scan *CrysAlis RED*; Oxford Diffraction, 2008)	*k* = −12→12
*T*_min_ = 0.919, *T*_max_ = 0.939	*l* = −16→18
14782 measured reflections	

### Refinement

**Table:**

Refinement on *F*^2^	Primary atom site location: structure-invariant direct methods
Least-squares matrix: full	Secondary atom site location: difference Fourier map
*R*[*F*^2^ > 2σ(*F*^2^)] = 0.050	Hydrogen site location: inferred from neighbouring sites
*wR*(*F*^2^) = 0.149	H atoms treated by a mixture of independent and constrained refinement
*S* = 0.98	*w* = 1/[σ^2^(*F*_o_^2^) + (0.0834*P*)^2^] where *P* = (*F*_o_^2^ + 2*F*_c_^2^)/3
4709 reflections	(Δ/σ)_max_ < 0.001
199 parameters	Δρ_max_ = 0.42 e Å^−^^3^
0 restraints	Δρ_min_ = −0.48 e Å^−^^3^

### Special details

**Table:**

Geometry. All esds (except the esd in the dihedral angle between two l.s. planes) are estimated using the full covariance matrix. The cell esds are taken into account individually in the estimation of esds in distances, angles and torsion angles; correlations between esds in cell parameters are only used when they are defined by crystal symmetry. An approximate (isotropic) treatment of cell esds is used for estimating esds involving l.s. planes.
Refinement. Refinement of *F*^2^ against ALL reflections. The weighted *R*-factor *wR* and goodness of fit *S* are based on *F*^2^, conventional *R*-factors *R* are based on *F*, with *F* set to zero for negative *F*^2^. The threshold expression of *F*^2^ > 2σ(*F*^2^) is used only for calculating *R*-factors(gt) *etc*. and is not relevant to the choice of reflections for refinement. *R*-factors based on *F*^2^ are statistically about twice as large as those based on *F*, and *R*-factors based on ALL data will be even larger.

### Fractional atomic coordinates and isotropic or equivalent isotropic displacement parameters (Å^2^)

**Table:**

	*x*	*y*	*z*	*U*_iso_*/*U*_eq_	
S1	0.06634 (4)	0.07477 (6)	0.41647 (4)	0.07016 (19)	
N1	0.23358 (10)	0.04869 (14)	−0.03349 (11)	0.0461 (3)	
H1A	0.1893 (14)	0.1126 (19)	−0.0463 (14)	0.055*	
N2	0.34252 (10)	−0.14141 (14)	0.08645 (10)	0.0444 (3)	
H2A	0.3614 (13)	−0.1995 (19)	0.1435 (14)	0.053*	
C1	0.28153 (11)	0.03270 (15)	−0.11059 (12)	0.0402 (3)	
C2	0.25289 (13)	0.11684 (18)	−0.20761 (13)	0.0509 (4)	
H2	0.1989	0.1861	−0.2242	0.061*	
C3	0.30433 (14)	0.09910 (17)	−0.28158 (13)	0.0534 (4)	
H3	0.2833	0.1558	−0.3476	0.064*	
C4	0.38422 (13)	0.00088 (17)	−0.25892 (13)	0.0485 (4)	
H4	0.4181	−0.0077	−0.3087	0.058*	
C5	0.49989 (12)	−0.19240 (16)	−0.13262 (12)	0.0447 (3)	
H5	0.5358	−0.2034	−0.1803	0.054*	
C6	0.52808 (12)	−0.27612 (16)	−0.03688 (12)	0.0461 (3)	
H6	0.5829	−0.3440	−0.0203	0.055*	
C7	0.47646 (12)	−0.26238 (16)	0.03701 (12)	0.0441 (3)	
H7	0.4970	−0.3210	0.1020	0.053*	
C8	0.39514 (11)	−0.16235 (14)	0.01405 (11)	0.0382 (3)	
C9	0.36357 (11)	−0.07342 (14)	−0.08561 (11)	0.0366 (3)	
C10	0.41675 (11)	−0.08885 (14)	−0.16042 (11)	0.0393 (3)	
C11	0.21019 (11)	−0.00769 (16)	0.15450 (12)	0.0417 (3)	
C12	0.25773 (12)	−0.05279 (17)	0.26845 (13)	0.0485 (4)	
H12	0.3227	−0.1011	0.2935	0.058*	
C13	0.21171 (13)	−0.02803 (18)	0.34438 (14)	0.0505 (4)	
H13	0.2462	−0.0587	0.4196	0.061*	
C14	0.11401 (11)	0.04254 (16)	0.31007 (13)	0.0444 (3)	
C15	0.06397 (11)	0.08679 (16)	0.19740 (13)	0.0454 (3)	
H15	−0.0018	0.1327	0.1724	0.054*	
C16	0.11204 (11)	0.06258 (16)	0.12205 (13)	0.0448 (3)	
H16	0.0777	0.0943	0.0470	0.054*	
C17	−0.05682 (14)	0.1664 (2)	0.34505 (16)	0.0674 (5)	
H17A	−0.0453	0.2571	0.3128	0.101*	
H17B	−0.0882	0.1875	0.3984	0.101*	
H17C	−0.1034	0.1042	0.2854	0.101*	
B1	0.26262 (13)	−0.03403 (18)	0.06810 (14)	0.0412 (4)	

### Atomic displacement parameters (Å^2^)

**Table:**

	*U*^11^	*U*^22^	*U*^33^	*U*^12^	*U*^13^	*U*^23^
S1	0.0687 (3)	0.0942 (4)	0.0586 (3)	0.0245 (3)	0.0370 (2)	0.0095 (2)
N1	0.0447 (7)	0.0488 (7)	0.0469 (7)	0.0105 (5)	0.0205 (6)	0.0060 (6)
N2	0.0530 (7)	0.0436 (7)	0.0412 (7)	0.0061 (5)	0.0237 (6)	0.0095 (5)
C1	0.0423 (7)	0.0380 (7)	0.0377 (7)	−0.0020 (6)	0.0133 (6)	−0.0009 (5)
C2	0.0560 (9)	0.0479 (9)	0.0456 (8)	0.0103 (7)	0.0167 (7)	0.0085 (7)
C3	0.0677 (11)	0.0499 (9)	0.0391 (8)	0.0042 (8)	0.0174 (7)	0.0095 (6)
C4	0.0664 (10)	0.0447 (8)	0.0380 (8)	−0.0024 (7)	0.0245 (7)	−0.0008 (6)
C5	0.0523 (9)	0.0437 (8)	0.0417 (8)	−0.0027 (6)	0.0226 (7)	−0.0071 (6)
C6	0.0487 (8)	0.0412 (8)	0.0477 (8)	0.0056 (6)	0.0182 (7)	−0.0043 (6)
C7	0.0527 (9)	0.0390 (7)	0.0399 (7)	0.0046 (6)	0.0177 (7)	0.0038 (6)
C8	0.0430 (7)	0.0346 (7)	0.0369 (7)	−0.0035 (5)	0.0158 (6)	−0.0015 (5)
C9	0.0405 (7)	0.0334 (7)	0.0331 (6)	−0.0058 (5)	0.0115 (5)	−0.0041 (5)
C10	0.0465 (8)	0.0356 (7)	0.0356 (7)	−0.0073 (6)	0.0163 (6)	−0.0066 (5)
C11	0.0407 (7)	0.0415 (7)	0.0438 (8)	−0.0018 (6)	0.0179 (6)	−0.0002 (6)
C12	0.0443 (8)	0.0541 (9)	0.0484 (8)	0.0107 (7)	0.0196 (7)	0.0055 (7)
C13	0.0499 (9)	0.0579 (9)	0.0445 (8)	0.0091 (7)	0.0196 (7)	0.0068 (7)
C14	0.0447 (8)	0.0415 (8)	0.0500 (8)	−0.0011 (6)	0.0220 (7)	−0.0008 (6)
C15	0.0368 (7)	0.0488 (8)	0.0488 (8)	0.0026 (6)	0.0152 (6)	0.0010 (6)
C16	0.0409 (7)	0.0499 (8)	0.0407 (8)	−0.0019 (6)	0.0132 (6)	0.0012 (6)
C17	0.0618 (11)	0.0654 (11)	0.0844 (13)	0.0100 (9)	0.0391 (10)	−0.0003 (10)
B1	0.0405 (8)	0.0420 (9)	0.0414 (8)	−0.0037 (6)	0.0166 (7)	−0.0013 (6)

### Geometric parameters (Å, °)

**Table:**

S1—C14	1.7534 (15)	C6—H6	0.9300
S1—C17	1.7789 (18)	C7—C8	1.3775 (19)
N1—C1	1.3944 (18)	C7—H7	0.9300
N1—B1	1.412 (2)	C8—C9	1.4213 (18)
N1—H1A	0.808 (17)	C9—C10	1.4231 (18)
N2—C8	1.3961 (17)	C11—C16	1.398 (2)
N2—B1	1.416 (2)	C11—C12	1.400 (2)
N2—H2A	0.852 (17)	C11—B1	1.559 (2)
C1—C2	1.373 (2)	C12—C13	1.372 (2)
C1—C9	1.4188 (19)	C12—H12	0.9300
C2—C3	1.398 (2)	C13—C14	1.393 (2)
C2—H2	0.9300	C13—H13	0.9300
C3—C4	1.353 (2)	C14—C15	1.386 (2)
C3—H3	0.9300	C15—C16	1.385 (2)
C4—C10	1.414 (2)	C15—H15	0.9300
C4—H4	0.9300	C16—H16	0.9300
C5—C6	1.359 (2)	C17—H17A	0.9600
C5—C10	1.411 (2)	C17—H17B	0.9600
C5—H5	0.9300	C17—H17C	0.9600
C6—C7	1.3948 (19)		
			
C14—S1—C17	104.67 (8)	C1—C9—C10	119.39 (12)
C1—N1—B1	123.67 (13)	C8—C9—C10	119.41 (12)
C1—N1—H1A	117.5 (12)	C5—C10—C4	122.98 (13)
B1—N1—H1A	118.8 (12)	C5—C10—C9	118.55 (12)
C8—N2—B1	123.82 (12)	C4—C10—C9	118.48 (13)
C8—N2—H2A	115.0 (11)	C16—C11—C12	116.00 (13)
B1—N2—H2A	121.2 (11)	C16—C11—B1	121.58 (13)
C2—C1—N1	122.28 (13)	C12—C11—B1	122.42 (13)
C2—C1—C9	119.75 (13)	C13—C12—C11	122.17 (14)
N1—C1—C9	117.96 (12)	C13—C12—H12	118.9
C1—C2—C3	120.43 (14)	C11—C12—H12	118.9
C1—C2—H2	119.8	C12—C13—C14	120.76 (14)
C3—C2—H2	119.8	C12—C13—H13	119.6
C4—C3—C2	121.17 (14)	C14—C13—H13	119.6
C4—C3—H3	119.4	C15—C14—C13	118.60 (13)
C2—C3—H3	119.4	C15—C14—S1	124.99 (11)
C3—C4—C10	120.75 (14)	C13—C14—S1	116.35 (12)
C3—C4—H4	119.6	C16—C15—C14	119.96 (13)
C10—C4—H4	119.6	C16—C15—H15	120.0
C6—C5—C10	120.69 (13)	C14—C15—H15	120.0
C6—C5—H5	119.7	C15—C16—C11	122.50 (14)
C10—C5—H5	119.7	C15—C16—H16	118.7
C5—C6—C7	121.36 (14)	C11—C16—H16	118.7
C5—C6—H6	119.3	S1—C17—H17A	109.5
C7—C6—H6	119.3	S1—C17—H17B	109.5
C8—C7—C6	120.23 (13)	H17A—C17—H17B	109.5
C8—C7—H7	119.9	S1—C17—H17C	109.5
C6—C7—H7	119.9	H17A—C17—H17C	109.5
C7—C8—N2	122.63 (12)	H17B—C17—H17C	109.5
C7—C8—C9	119.77 (12)	N1—B1—N2	115.72 (13)
N2—C8—C9	117.58 (12)	N1—B1—C11	121.92 (13)
C1—C9—C8	121.19 (12)	N2—B1—C11	122.36 (13)
			
B1—N1—C1—C2	−178.71 (15)	C1—C9—C10—C5	178.43 (12)
B1—N1—C1—C9	0.4 (2)	C8—C9—C10—C5	−0.22 (18)
N1—C1—C2—C3	178.83 (14)	C1—C9—C10—C4	−1.23 (19)
C9—C1—C2—C3	−0.3 (2)	C8—C9—C10—C4	−179.88 (12)
C1—C2—C3—C4	−1.0 (2)	C16—C11—C12—C13	−0.8 (2)
C2—C3—C4—C10	1.2 (2)	B1—C11—C12—C13	179.34 (14)
C10—C5—C6—C7	−0.2 (2)	C11—C12—C13—C14	0.7 (2)
C5—C6—C7—C8	−0.1 (2)	C12—C13—C14—C15	0.2 (2)
C6—C7—C8—N2	−178.36 (13)	C12—C13—C14—S1	−177.34 (12)
C6—C7—C8—C9	0.3 (2)	C17—S1—C14—C15	2.01 (16)
B1—N2—C8—C7	176.50 (14)	C17—S1—C14—C13	179.41 (13)
B1—N2—C8—C9	−2.2 (2)	C13—C14—C15—C16	−1.0 (2)
C2—C1—C9—C8	−179.98 (13)	S1—C14—C15—C16	176.34 (11)
N1—C1—C9—C8	0.86 (19)	C14—C15—C16—C11	0.9 (2)
C2—C1—C9—C10	1.4 (2)	C12—C11—C16—C15	0.0 (2)
N1—C1—C9—C10	−177.77 (12)	B1—C11—C16—C15	179.86 (13)
C7—C8—C9—C1	−178.74 (12)	C1—N1—B1—N2	−2.4 (2)
N2—C8—C9—C1	−0.03 (19)	C1—N1—B1—C11	177.28 (13)
C7—C8—C9—C10	−0.11 (19)	C8—N2—B1—N1	3.3 (2)
N2—C8—C9—C10	178.60 (12)	C8—N2—B1—C11	−176.37 (13)
C6—C5—C10—C4	−179.96 (14)	C16—C11—B1—N1	20.3 (2)
C6—C5—C10—C9	0.4 (2)	C12—C11—B1—N1	−159.84 (15)
C3—C4—C10—C5	−179.68 (14)	C16—C11—B1—N2	−160.00 (14)
C3—C4—C10—C9	0.0 (2)	C12—C11—B1—N2	19.8 (2)

### Hydrogen-bond geometry (Å, °)

**Table:**

*D*—H···*A*	*D*—H	H···*A*	*D*···*A*	*D*—H···*A*
C2—H2···Cg3^i^	0.93	2.75	3.5178 (18)	141
C12—H12···Cg2^ii^	0.93	2.82	3.6122 (17)	144

Symmetry codes: (i) *x*, −*y*−1/2, *z*−3/2; (ii) *x*, −*y*−3/2, *z*−1/2.

## References

[bb1] Farrugia, L. J. (1999). *J. Appl. Cryst.* **32**, 837–838.

[bb2] Oxford Diffraction (2008). *CrysAlis CCD* and *CrysAlis RED* Oxford Diffraction Ltd, Abingdon, Oxfordshire, England.

[bb3] Sheldrick, G. M. (2008). *Acta Cryst.* A**64**, 112–122. 10.1107/S0108767307043930 18156677

[bb4] Slabber, C. A. (2011). *Ultrastabilized Boranes: A Study into the Synthesis, Structure and Reactivities of Heterosubstituted Organoboranes* MSc Thesis, University of KwaZulu Natal, South Africa.

[bb5] Spek, A. L. (2009). *Acta Cryst.* D**65**, 148–155. 10.1107/S090744490804362X PMC2631630 19171970

[bb6] Weber, L., Werner, V., Fox, M. A., Marder, R. T. S., Schwedler, S., Brockhinke, A., Stammler, H.-G. & Neumann, B. (2009). *Dalton Trans.* pp. 1339–1351. 10.1039/b815931a 19462655

